# The Presynaptic Protein Mover Is Differentially Expressed Across Brain Areas and Synapse Types

**DOI:** 10.3389/fnana.2018.00058

**Published:** 2018-07-13

**Authors:** Rebecca Wallrafen, Thomas Dresbach

**Affiliations:** Synaptogenesis Group, Institute of Anatomy and Embryology, University Medical Center Goettingen, Goettingen, Germany

**Keywords:** mover, synaptic protein, distribution, immunofluorescence, quantification, hippocampus, amygdala

## Abstract

The assembly and function of presynaptic nerve terminals relies on evolutionarily conserved proteins. A small number of presynaptic proteins occurs only in vertebrates. These proteins may add specialized functions to certain synapses, thus increasing synaptic heterogeneity. Here, we show that the vertebrate-specific synaptic vesicle (SV) protein mover is differentially distributed in the forebrain and cerebellum of the adult mouse. Using a quantitative immunofluorescence approach, we compare the expression of mover to the expression of the general SV marker synaptophysin in 16 brain areas. We find that mover is particularly abundant in the septal nuclei (SNu), ventral pallidum (VPa), amygdala and hippocampus. Within the hippocampus, mover is predominantly associated with excitatory synapses. Its levels are low in layers that receive afferent input from the entorhinal cortex, and high in layers harboring intra-hippocampal circuits. In contrast, mover levels are high in all nuclei of the amygdala, and mover is associated with inhibitory synapses in the medioposterior amygdala. Our data reveal a striking heterogeneity in the abundance of mover on three levels, i.e., between brain areas, within individual brain areas and between synapse types. This distribution suggests a role for mover in providing specialization to subsets of synapses, thereby contributing to the functional diversity of brain areas.

## Introduction

Neurotransmitter release is mediated by a molecular machinery consisting of proteins mediating synaptic vesicle (SV) anchoring, priming and fusion at specialized sites of presynaptic nerve terminals called active zones (Fejtova and Gundelfinger, 2006; Südhof, 2012). The vast majority of presynaptic proteins is evolutionarily conserved, but a remarkably small number of proteins is unique to vertebrates. These include the active zone scaffolding proteins bassoon and piccolo, the motor adaptor syntabulin, and the SV proteins synuclein and mover (George, 2002; Cai et al., 2007; Kremer et al., 2007; Gundelfinger et al., 2016). It has been suggested that the major role of vertebrate-specific synaptic proteins is to increase the functional heterogeneity of synapses in the brain (Emes et al., 2008; Ryan and Grant, 2009). Numerous examples have demonstrated heterogeneous release probability and short-term plasticity among synapses in the neocortex, hippocampus and cerebellum (Blackman et al., 2013). While some of this heterogeneity may arise from different combinations of isoforms and posttranslational modifications of the conserved core machinery, vertebrate-specific proteins may add additional versatility to this machinery. In particular, cell- or synapse-specific expression of such proteins may endow certain synapses with special features.

Bassoon is a vertebrate-specific component of active zones that is found at all synapses and may generally stabilize presynaptic boutons by reducing proteasomal degradation (Waites et al., 2013; Okerlund et al., 2017). Bassoon interacts with—among a number of evolutionarily conserved proteins—two of the vertebrate-specific proteins, i.e., syntabulin (Cai et al., 2007) and mover (Kremer et al., 2007). We had identified mover as a binding partner for bassoon in a yeast-2-hybrid assay and found that it is a phosphoprotein of SVs (Kremer et al., 2007; Ahmed et al., 2013). Knockdown of mover at the calyx of Held synapse resulted in increased release probability and short-term depression, suggesting that mover regulates synaptic strength and plasticity at this synapse (Körber et al., 2015). Mover has also been detected in a proteomic analysis of SV fractions, where it was called SVAP-30 (Burré et al., 2006). In addition, mover is called TPRGL. It appears to have co-evolved with a similar protein called TPRG by gene duplication in vertebrates, and mover/TPRGL and TPRG are each located next to a vertebrate-specific transcription factor, called p73 and p63, respectively (Antonini et al., 2008).

Interestingly, unlike bassoon, mover appeared to be absent from some synapses: on a qualitative level, we had detected mover in hippocampal mossy fiber terminals and at the calyx of Held, i.e., two glutamatergic synapses, and at inhibitory synapses in the cerebellum. In contrast, we had not detected mover at inhibitory terminals in the stratum lucidum of the hippocampus (Kremer et al., 2007). To test whether mover is indeed a candidate protein that could increase the functional heterogeneity of synapses we tested its distribution on a quantitative level, employing a comparative approach relating mover expression levels to the abundance of SVs.

## Materials and Methods

### Experimental Animals

No experiments involving live animals were conducted for this study. Experiments involving euthanizing of animals to obtain brain samples were approved by the local animal protection authorities (Tierschutzkommission der Universitätsmedizin Göttingen) under the approval number T 10/30.

### Immunofluorescence Staining

For this study, three adult male wild-type (wt) C57BL/6 mice were euthanized by CO_2_ inhalation and transcardially perfused with 0.9% saline followed by perfusion with 4% paraformaldehyde (PFA) in 0.1 M phosphate buffer (PB), pH 7.4. Brains were removed and postfixed in 4% PFA in 0.1 M PB for 24 h at 4°C and incubated in 30% sucrose at 4°C for 48 h.

Brains were cut into 25 μm thick coronal sections using a freezing microtome. The sections were collected in 0.1 M PB and stored at 4°C until further use. Five positions relative to Bregma were selected according to the mouse brain atlas (Paxinos and Franklin, 2001), and per level, three adjacent slices per brain were stained (Bregma ranges: +1.5 mm, +1.0 mm, −2.0 mm, −3.5 mm, −6.0 mm). Note that therefore quantitative results apply to the indicated Bregma levels. Free floating sections were rinsed with PB once and blocked with 2.5% goat serum (Merck KGaA, Darmstadt, Germany), 2.5% donkey serum (Merck Chemikals GmbH, Darmstadt, Germany), 1% Triton X-100 in PB for 3 h at room temperature (RT). The following relevant primary antibodies were applied overnight at 4°C: mover (1:1000 rabbit anti-mover polyclonal, Cat. No. 248003, RRID:AB_10804285, Synaptic Systems, Goettingen, Germany), synaptophysin (1:1000 guinea pig anti-synaptophysin polyclonal, Cat. No. 101004, RRID:AB_1210382, Synaptic Systems, Goettingen, Germany), vGlut1 (1:1000 guinea pig anti vGluT1 polyclonal, Cat. No. 135304, RRID:AB_887878, Synaptic Systems, Goettingen, Germany) and vGAT (1:500 chicken anti-vGAT polyclonal, Cat. No. 131006, RRID:AB_2619820, Synaptic Systems, Goettingen, Germany). The sections were washed with 2% goat serum in PB and incubated with relevant secondary antibodies for 90 min at RT in the dark: donkey anti-rabbit 647 (1:1000, Alexa Fluor, Invitrogen, Carlsbad, CA, USA), goat anti-guinea pig Cy2, goat anti-chicken Cy3 (1:1000, Jackson ImmunoResearch, West Grove, PA, USA; all antibodies were diluted in 0.5% goat serum, 0.5% donkey serum, 0.2% Triton X-100 in PB). The sections were washed with 1% goat serum in PB, incubated with 4′,6-diamidino-2-phenylindole (DAPI; 1:1000 in PB) for 5 min, rinsed again and mounted on Menzel microscope slides. To ensure minimal variability, brains from all animals were cut, stained and treated simultaneously.

### Microscopy

All images were acquired using a Zeiss LSM800 confocal microscope, running the ZEN blue software (version 2.3, Zeiss, Oberkochen, Germany). Laser settings were adjusted so that few pixels were overexposed to ensure maximum distribution of gray values.

#### Distribution Analysis

To analyze the distribution of mover throughout the brain, double stainings for mover and synaptophysin were performed and virtual tissues composed of single tiles (1024 × 1024 px, moderate scan speed, four-times averaging) were acquired using a 10× objective (air immersion, NA 0.45). The whole brain slice was imaged. Using the corresponding functions of the program, virtual tissues were stitched and exported as TIFF-files.

#### Colocalization Analysis

To determine the colocalization of mover with vGluT1 and vGAT, triple stainings were performed and single pictures in the ROI were acquired using a 40× objective (oil immersion, NA 1.3). No adjustments for brightness or contrast were made, and images were exported as TIFF-files.

### Quantification

For quantification, areas of interest were delineated manually using FIJI (ImageJ v.1.51r) with the mouse brain atlas as reference. First, mean fluorescence intensity (MFI) values were determined for one area of interest for the synaptophysin and mover channels. These values were transferred to Microsoft Excel for data handling. To determine their ratio, the value from the mover channel was divided by the value from the synaptophysin channel. Second, MFI values were determined for all pixels across the entire image of the hemisphere (including pixels from areas of interest and the remaining pixels). The ratio in one area of interest was then compared to the ratio in the corresponding hemisphere. We performed these actions for every brain region and slice separately, i.e., always comparing the ratio of mover vs. synaptophysin in the area of interest to the ratio of mover vs. synaptophysin in the hemisphere on the same slice. If the ratio in one brain region of interest and in the hemisphere were the same, the resulting ratio would be 1. Next, we determined how much the ratio in one brain area differs from the ratio in the hemisphere. We calculated the percentage that the ratio of mover vs. synaptophysin in the area of interest to the ratio of mover vs. synaptophysin in the hemisphere differed from 1. We refer to this percentage as the relative abundance of mover. For visualization, values are displayed in bar charts with individual values superimposed (indicating average ± SEM, GraphPrism 6).

### Colocalization Analysis

To analyze the colocalization of mover with vGluT1 and vGAT, respectively, we subtracted the background staining from the monochromatic image using the “Subtract Background” function of FIJI (Rolling ball radius: 100 pixels for all channels). We then used the “Colocalization Test” plugin to determine the Pearson’s correlation coefficient between mover and vGluT1, between mover and vGAT and vGluT1 and vGAT as a means of control (for more information on Pearson’s correlation see Adler and Parmryd, 2010 and Dunn et al., 2011). To verify that no random colocalization was measured, we rotated one of the images by 90° and analyzed colocalization: doing this, no colocalization was observed, and Pearson’s correlation values were very low to negative. Values were plotted in scatter plots including bar charts (average ± SEM) and a Student’s *t-test* was performed using GraphPrism 6.

### Antibody Specificity

To verify the absence of non-specific immunostaining using the immunofluorescence method, primary antibodies were excluded but the secondary antibody steps were performed to completion. Under these conditions, no cross-reactivity or significant background staining was observed (not shown).

## Results

### Mover Is Heterogeneously Expressed Throughout the Adult Mouse Brain

**Figure 1:**
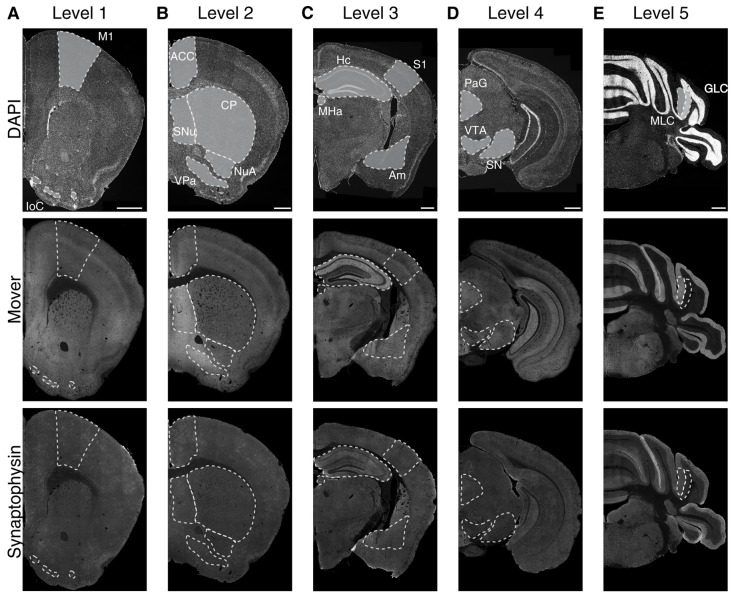
Immunofluorescence images of 4′,6-diamidino-2-phenylindole (DAPI), mover and synaptophysin at the five rostro-caudal levels. We examined five coronal levels of the mouse brain (Levels 1–5, **A–E**) and manually delineated 16 brain regions of interest (delineated with white dotted lines). The upper panels show the DAPI counterstaining, displaying the general anatomy at the plane of sectioning. Note the heterogeneous distribution of mover throughout the levels (middle panels), while the distribution of synaptophysin (lower panels) is rather homogeneous. M1, primary motor cortex; IoC, islands of Calleja; ACC, anterior cingulate cortex; SNu, septal nuclei; VPa, ventral pallidum; NuA, nucleus accumbens; CP, caudate putamen; S1, primary somatosensory cortex; Hc, hippocampus; Am, amygdala; MHa, medial habenula; PAG, periaqueductal gray; SN, substantia nigra; VTA, ventral tegmental area; MLC, molecular layer of the cerebellum; GLC, granular layer of the cerebellum. Scale bar = 500 μm.

To determine the regional distribution of the synaptic protein mover, we performed immunofluorescence double-stainings on five rostro-caudal levels for mover and synaptophysin, an integral membrane protein of SVs in all synapses (Navone et al., 1986), and counterstained the slices with DAPI (Figure 1, upper panels). Upon inspection, mover seemed heterogeneously distributed (Figure 1, middle panels), with areas of obviously high signal intensity, while synaptophysin signals were fairly constant across all areas of the hemispheres (Figure 1, lower panels). We selected 16 brain regions for quantification of mover: primary motor cortex (M1), islands of Calleja (IoC), anterior cingulate cortex (ACC), septal nuclei (SNu), ventral pallidum (VPa), nucleus accumbens (NuA), caudate and putamen (CP), primary somatosensory cortex (S1), hippocampus (Hc), amygdala (Am), medial habenula (MHa), periaqueductal gray (PAG), substantia nigra (SN), ventral tegmental area (VTA), molecular layer of the cerebellum (MLC) and granular layer of the cerebellum (GLC). Mover immunofluorescence intensities were particularly high in the SNu, the VPa and the amygdala, and strikingly low in the GLC, the MHa and the S1.

The difference in mover immunofluorescence intensities between the areas could reflect either of two scenarios: (a) areas with increased mover immunofluorescence intensities have synapses with an increased concentration of mover per SV; (b) areas with increased mover immunofluorescence intensities have synapses with more SVs. To test whether some synapses have a higher concentration of mover than others, we set out to quantify our observation. To this end, a marker representing the number of SVs per synapse has to be introduced. In our study, the integral SV protein synaptophysin represents this parameter.

**Figure 2:**
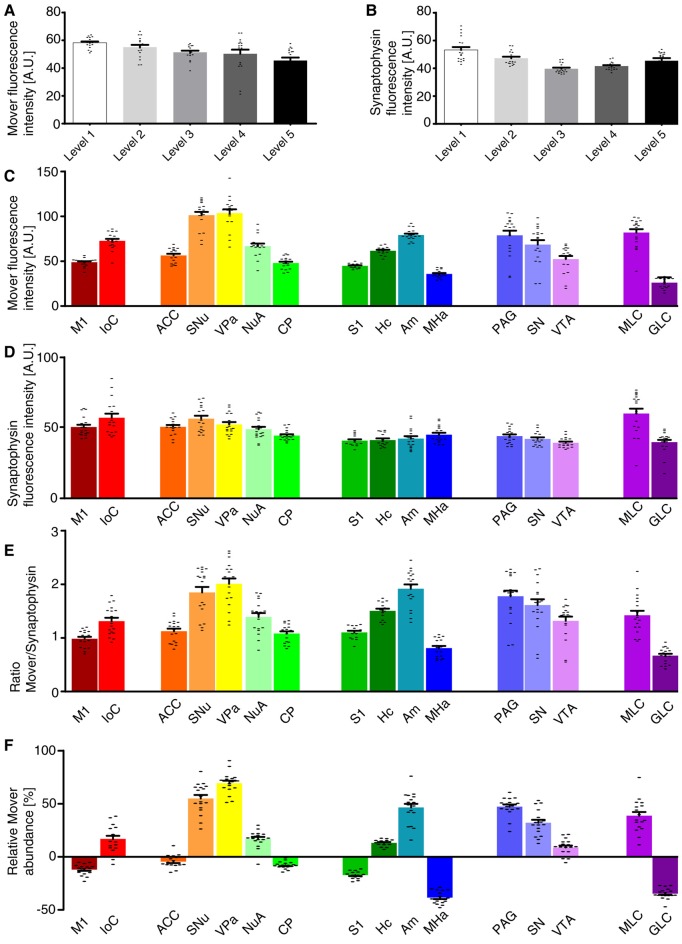
Quantification of the mover distribution across the five rostro-caudal levels.** (A,B)** Mean fluorescence intensity (MFI) of the mover signal **(A)** and the synaptophysin signal **(B)** at the different levels. **(C,D)** MFI of the mover signal **(C)** and the synaptophysin signal **(D)** at the 16 manually delineated brain regions. **(E)** Ratios of mover and synaptophysin in the different brain regions. **(F)** Quantification comparing mover/synaptophysin ratio at the respective region to the ratio of the whole hemisphere. Mover fluorescence is above average in islands of Calleja, but below average in M1. Mover levels are above average in the SNu, VPa and nucleus accumbens and below average in the ACC and caudate putamen. Mover levels are above average in the hippocampus and amygdala and below average in S1 and the medial habenula (MHa). Mover levels are above average in the periaqueductal gray, SN and VTA. Mover levels are above average in the MLC and below average in the granular layer of cerebellum. M1, primary motor cortex; IoC, islands of Calleja; ACC, anterior cingulate cortex; SNu, septal nuclei; VPa, ventral pallidum; NuA, nucleus accumbens; CP, caudate putamen; S1, primary somatosensory cortex; Hc, hippocampus; Am, amygdala; MHa, medial habenula; PAG, periaqueductal gray; SN, substantia nigra; VTA, ventral tegmental area; MLC, molecular layer of the cerebellum; GLC, granular layer of the cerebellum.

We first determined the MFI across the whole hemisphere for the two different channels, i.e., mover (Figure 2A) and synaptophysin (Figure 2B). For the 16 manually delineated brain regions we also determined the MFI of mover (Figure 2C) and synaptophysin (Figure 2D). Calculating their ratio (Figure 2E) revealed regions with high mover abundance relative to synaptophysin (like the VPa, the SNu and the amygdala) and regions with lower mover abundance (like the MHa, the GLC and the M1). At Bregma +1.5 mm, the relative abundance of mover was above average of the hemisphere in islands of Calleja (16.89 ± 2.83%, red), and below average of the hemisphere in M1, where it was heterogeneously distributed throughout the layers (−12.03 ± 1.12%, dark red). At Bregma +1.0 mm, mover abundance was above average in SNu, VPa and nucleus accumbens (55.02 ± 3.25%, orange; 69.45 ± 2.28%, yellow; and 16.91 ± 1.81%, mint green, respectively), and below average in ACC and caudate putamen (−4.51 ± 1.34%, dark orange; and −7.99 ± 0.75%, bright green). More caudally, at Bregma −2.0 mm, high mover levels were detected in hippocampus and amygdala (13.13 ± 0.79%, dark green; and 46.57 ± 3.31%, petrol), while low levels were detected in S1 and MHa (−17.26 ± 0.85%, green; and −38.41 ± 1.35%, dark blue). In the S1, like in the M1, we noticed a heterogeneous layer-related distribution of mover. While relative mover abundance overall was below average, some cortical layers, i.e., layer I and layer V, showed higher mover intensity than other layers such as layer IV (Figure 1C, middle panel). At Bregma −3.5 mm, mover was above average in all brain regions, i.e., periaqueductal gray, SN and VTA (47.31 ± 1.99%, light blue; 32.11 ± 2.71%, light purple; and 8.81 ± 1.74%, purple, respectively), while at the caudal-most level (Bregma −6.0 mm), mover levels were again heterogeneous, being high in the molecular layer (38.75 ± 3.41%, plum) and low in the GLC (−34.68 ± 1.13%, dark purple; Figure 2F). Overall, quantification revealed areas of increased and areas of decreased ratios of mover to synaptophysin compared to the average across the hemisphere. This corroborates the hypothesis that mover is differentially distributed throughout the adult mouse brain, and prompted us to investigate the pattern of mover in more detail within individual brain regions.

### Mover Is Differentially Distributed in the Different Layers of the Hippocampus

**Figure 3:**
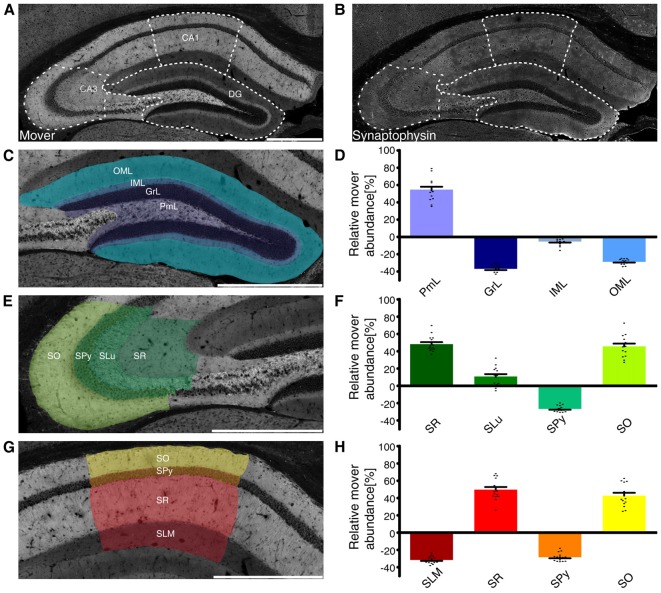
Mover distribution in the mouse hippocampus. Immunofluorescence staining of coronal slices of the mouse hippocampus. **(A,B)** Overview of the hippocampus showing the heterogeneous mover expression pattern **(A)** and the corresponding synaptophysin staining **(B)**. The three regions of interest (DG, **(C)**; CA3 **(E)**; CA1 **(G)**) are delineated with white dotted lines. **(D,F,H)** Quantification comparing the ratio in the respective layer to the ratio of the whole hemisphere. Mover is especially abundant in the polymorph layer of DG (purple), stratum radiatum, lucidum and oriens of CA3 (dark green/green/neon green) and CA1(red/yellow). Levels of mover below average are detected in the granular, inner and outer molecular layer of DG (dark blue/gray/blue), SPy of CA3 (light green) and CA1(orange) and stratum lacunosum-moleculare (dark red). OML, outer molecular layer; IML, inner molecular layer; GrL, granular layer; PmL, polymorph layer; SO, stratum oriens; SPy, stratum pyramidale; SLu, stratum lucidum; SR, stratum radiatum; SLM, stratum lacunosum-moleculare. Scale bar = 500 μm.

When analyzing mover abundance in the different brain regions, we were surprised by the relatively low value obtained for the hippocampus: while mover staining seemed especially bright in that region, quantification yielded a value around 13% above average. We also noted that mover was especially abundant in some layers of the hippocampus, while it seemed absent in others. We therefore determined mover-to-synaptophysin ratios in the different subregions and layers (Förster et al., 2006). While mover distribution was very heterogeneous throughout the hippocampus (Figure 3A), synaptophysin levels varied less, with higher levels only in the big mossy fiber terminals in the polymorph layer of DG, also called the hilus, and stratum lucidum of CA3, and lower levels in the stratum pyramidale (SPy; Figure 3B).

Analyzing the layers of the DG (Figure 3C), we saw high mover abundance in the polymorph layer (PmL 54.72 ± 2.90%, purple), which includes mossy fibers. In contrast, mover abundance was below average in the granular and outer molecular layer (GrL −36.93 ± 1.27% and OML −28.86 ± 0.71% respectively, dark blue/blue), while it was close to average in the inner molecular layer (IML −5.35 ± 1.02%, gray; Figure 3D).

In the CA3 region (Figure 3E), mover was highly abundant in the stratum radiatum and oriens (SR 48.19 ± 2.07% and SO 45.68 ± 2.79% respectively, dark green/neon green), while it was below average in the SPy (−26.67 ± 0.82%, light green). In the stratum lucidum, where the mossy fibers from DG granule cells terminate onto the apical dendrites of CA3 pyramidal cells, mover abundance was 10.75 ± 2.44% above average (SLu, green; Figure 3F).

A detailed analysis of the CA1 region (Figure 3G) yielded high mover ratios in the stratum radiatum, where Schaffer collaterals originating from CA3 pyramidal cells terminate onto pyramidal cells of CA1 (SR 49.70 ± 2.73%, red). In stratum oriens, mover abundance was also above average (SO 42.80 ± 2.88%, yellow), while both the stratum lacunosum-moleculare and pyramidale showed low levels of mover (SLM −31.61 ± 0.98% and SPy −28.43 ± 1.10% respectively, dark red/orange; Figure 3H). Thus, mover is specifically associated with stratum radiatum, stratum oriens and the polymorph layer of DG, while it is close to average in the inner molecular layer of DG, and strikingly below average in the cell body layers and stratum lacunosum-moleculare.

### Mover Is Present at Excitatory Synapses in the Hippocampus

**Figure 4:**
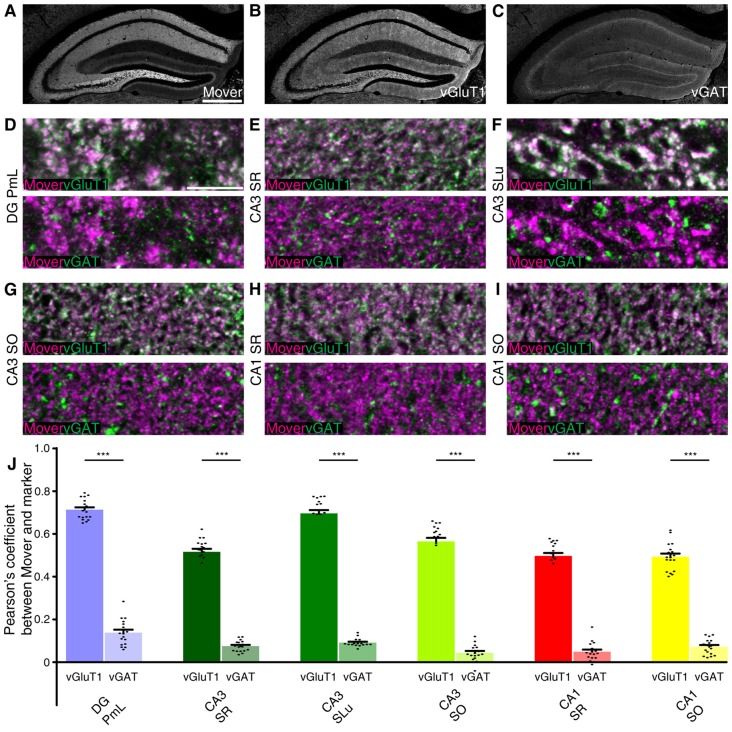
Mover colocalization with presynaptic markers vesicular glutamate transporter 1 (vGluT1) and vesicular γ-aminobutyric acid (GABA) transporter (vGAT) in the mouse hippocampus. Immunofluorescence triple labeling of the mouse hippocampus. **(A–C)** Overview showing the heterogeneous mover expression pattern **(A)** and the corresponding vGluT1 **(B)** and vGAT staining **(C)**. **(D–I)** Overlay of mover (magenta) and vGluT1 staining (green, upper panel) and mover (magenta) and vGAT (green, lower panel) in regions of the hippocampus where mover immunofluorescence was above average. **(D)** High colocalization of mover with vGluT1 in polymorph layer of DG, low colocalization of mover with vGAT. **(E)** High colocalization of mover with vGluT1 in stratum radiatum of CA3, low colocalization of mover with vGAT. **(F)** High colocalization of mover with vGluT1 in stratum lucidum of CA3, low colocalization of mover with vGAT. **(G)** High colocalization of mover with vGluT1 in stratum oriens of CA3, low colocalization of mover with vGAT. **(H)** High colocalization of mover with vGluT1 in stratum radiatum of CA1, low colocalization of mover with vGAT. **(I)** High colocalization of mover with vGluT1 in stratum oriens of CA1, low colocalization of mover with vGAT. **(J)** Visualization of the quantification of colocalization with the different markers using the Pearson’s correlation coefficient. Bars show average ± SEM; ***P < 0.001. PmL, polymorph layer of dentate gyrus; SR, stratum radiatum (of either CA3 or CA1); SLu, stratum lucidum of CA3; SO, stratum oriens (of either CA3 or CA1). Scale bar: **(A–C)** = 500 μm, **(D–I)** = 10 μm.

To test whether this distributional heterogeneity also applies to synapse types, we triple-stained the hippocampus. We used the vesicular glutamate transporter 1 (vGluT1) as a marker for excitatory synapses (Ziegler et al., 2002). As a marker for inhibitory nerve terminals we used the vesicular γ-aminobutyric acid (GABA) transporter (vGAT; Chaudhry et al., 1998). We focused on regions with high mover-to-synaptophysin ratios, and applied colocalization analysis using Pearson’s correlation coefficient as a read-out. If two markers colocalize perfectly, Pearson’s correlation coefficient would be 1; random distribution of signals or avoidance will yield low values, i.e., close to 0 or negative. As a means of quality check for our stainings, we performed the colocalization analysis between vGluT1 and vGAT, and detected low to very low Pearson’s correlation coefficients (DG PmL: 0.169, Supplementary Figure S1A; CA3 SR: 0.055, Supplementary Figure S1B; CA3 SLu: 0.103, Supplementary Figure S1C; CA3 SO: 0.027, Supplementary Figure S1D; CA1 SR: 0.052, Supplementary Figure S1E; CA1 SO: 0.074, Supplementary Figure S1F; comparison of all values Supplementary Figure S1G). The low Pearson’s correlation values corroborate the quality of the staining and make colocalization analysis between mover and the different markers feasible (Figure 4). The rather high values in polymorph layer of DG (0.169) and stratum lucidum of CA3 (0.103) can be explained by the complex intermingling of GABAergic synapses and the extraordinarily large, excitatory mossy fiber terminals found in these regions. We therefore assume that in these regions, even these relatively high values reflect a lack of colocalization.

Low magnification images of the individual channels of our triple stainings indicated similarities between the mover distribution (Figure 4A) and vGluT1 (Figure 4B). Accordingly, colocalization analysis of high magnification images yielded high values in all regions (DG PmL: 0.713, Figure 4D and Supplementary Figure S2A; CA3 SR: 0.516, Figure 4E and Supplementary Figure S2B; CA3 SLu: 0.696, Figure 4F and Supplementary Figure S2C; CA3 SO: 0.565, Figure 4G and Supplementary Figure S2D; CA1 SR: 0.497, Figure 4H and Supplementary Figure S2E; CA1 SO: 0.493, Figure 4I and Supplementary Figure S2F; comparison of all values Figure 4J). The distribution patterns of mover and vGAT (Figure 4C) differed substantially from each other, which was also reflected in the low Pearson’s correlation coefficient in all regions (DG PmL: 0.138, Figure 4D and Supplementary Figure S2A; CA3 SR: 0.075, Figure 4E and Supplementary Figure S2B; CA3 SLu: 0.091, Figure 4F and Supplementary Figure S2C; CA3 SO: 0.044, Figure 4G and Supplementary Figure S2D; CA1 SR: 0.049, Figure 4H and Supplementary Figure S2E; CA1 SO: 0.072, Figure 4I and Supplementary Figure S2F; comparison of all values Figure 4J). The Pearson’s correlation coefficient in DG PmL (0.138) did not exceed the value of vGluT1 and vGAT, and therefore likely indicated lack of colocalization. Colocalization between mover and vGluT1 was significantly higher than colocalization between mover and vGAT in all areas (all *P*-values were *P* <<< 0.0001). Taken together, these data suggest that in the hippocampus, mover is enriched at excitatory synapses, while it is absent from inhibitory synapses.

### Mover Is Enriched in the Different Amygdaloid Nuclei

**Figure 5:**
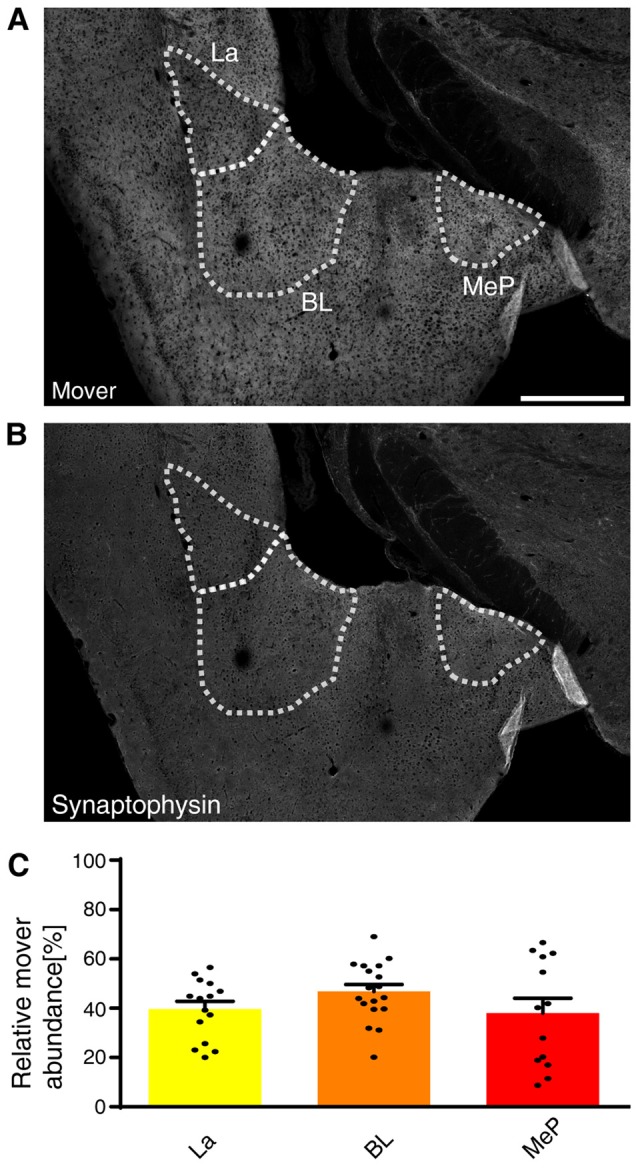
Mover distribution in the mouse amygdala. Immunofluorescence staining of coronal slices of the mouse amygdala. **(A,B)** Overview showing the heterogeneous mover expression pattern **(A)** and the corresponding synaptophysin staining **(B)**. The three regions of interest (La, BL, MeP) are delineated with white dotted lines. **(C)** Quantification comparing the ratio in the respective nuclei to the ratio of the whole hemisphere. High mover expression is detected in all three amygdaloid nuclei (La, yellow; BL, orange; MeP, red). Bars show average ± SEM. La, lateral nuclei of the amygdala; BL, basolateral nuclei of the amygdala; MeP, medioposterior nuclei of the amygdala. Scale bar = 500 μm.

Mover was even more abundant in the amygdala compared to the hippocampus (Figures 2E,F). Within the amygdala, mover and synaptophysin were homogeneously distributed among the different nuclei (Figures 5A,B). In the lateral amygdaloid nuclei (La), which receive glutamatergic input from sensory systems (Davis and Whalen, 2001), mover abundance was high (39.61 ± 2.75%, yellow, Figure 5C). The same held true for the basolateral amygdala (BL; 46.73 ± 2.75%, orange), which receives input mainly from the auditory system, the hippocampus and the prefrontal cortex (McDonald et al., 1996; Baars and Gage, 2010; McGarry and Carter, 2017), and medioposterior amygdaloid nuclei (MeP; 37.98 ± 4.92%, red; Figure 5C), which receives input from the main and accessory olfactory bulbs (Keshavarzi et al., 2014). In contrast to hippocampus, mover abundance was homogeneously high throughout the amygdala.

### Mover Is Present at Both Excitatory and Inhibitory Synapses in the Amygdala

**Figure 6:**
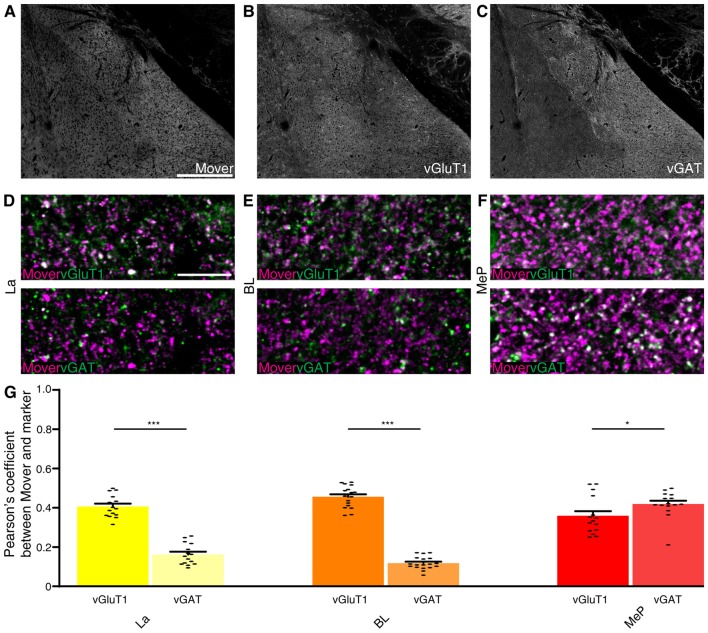
Mover colocalization with presynaptic markers vGlut1 and vGAT in the mouse amygdala. Immunofluorescence triple labeling of the mouse amygdala. **(A–C)** Overview showing the heterogeneous mover expression pattern **(A)** and the corresponding vGluT1 **(B)** and vGAT staining **(C)**. **(D–F)** Overlay of mover (magenta) and vGluT1 staining (Green, Upper Panel) and mover (magenta) and vGAT (green, lower panel) in the different nuclei of the amygdala. (**D**) Moderate colocalization of mover with vGlut1 in lateral amygdaloid nuclei, low colocalization of mover with vGAT. **(E)** Moderate colocalization of mover with vGlut1 in basolateral amygdaloid nuclei, low colocalization of mover with vGAT. **(F)** Moderate colocalization of mover with vGlut1 in medioposterior amygdaloid nuclei, moderate colocalization of mover with vGAT. **(G)** Visualization of the quantification of colocalization with the different markers using the Pearson’s coefficient. Bars show average ± SEM; *P < 0.05; ***P < 0.001. La, lateral nuclei of the amygdala; BL, basolateral nuclei of the amygdala; MeP, medioposterior nuclei of the amygdala. Scale bar: **(A–C)** = 500 μm, **(D–F)** = 10 μm.

As input into the amygdala is so diverse, we analyzed the colocalization of mover with vGluT1 and vGAT. Here again we checked for the Pearson’s correlation coefficient between vGluT1 and vGAT as a means of quality control and detected very low correlation values (La: 0.088, Supplementary Figure S3A; BL: 0.073, Supplementary Figure S3B; MeP: 0.074, Supplementary Figure S3C; comparison of all values Supplementary Figure S3D). Mover (Figure 6A) and vGluT1 (Figure 6B) colocalized in all three regions (La: 0.386, Figures 6D, Supplementary Figure S4A; BL: 0.456; Figure 6E and Supplementary Figure S4B, MeP: 0.359; Figure 6F and Supplementary Figure S4C; comparison of all values Figure 6G) while colocalization with vGAT (Figure 6C) was strong in medioposterior nuclei (0.419, Figure 6F and Supplementary Figure S4C), but weak in lateral (0.162, Figure 6D and Supplementary Figure S4A) and basolateral nuclei (0.140, Figure 6E and Supplementary Figure S4B). In the lateral and basolateral nuclei, colocalization between mover and vGlutT1 exceeded colocalization between mover and vGAT significantly (*P*-values: La: 2.72 × 10^−8^; BL: *P* <<< 0.0001), while in the medioposterior nucleus, colocalization between mover and vGAT was significantly higher than colocalization between mover and vGluT1 (*P*-value 0.043). Like in the hippocampus, mover is enriched at excitatory synapses throughout the amygdala. In contrast, synapses of the medioposterior nucleus display prominent mover staining. Overall, these data indicate that even though mover levels are high throughout the amygdala, mover is differentially distributed across synapse types in this brain region.

## Discussion

With this study, we provide the first quantitative description of the distribution of mover, a vertebrate-specific SV protein we identified as a binding partner for the presynaptic scaffolding molecule bassoon (Kremer et al., 2007; Ahmed et al., 2013). A qualitative description had suggested that mover might be present at some synapses and absent from others. Here, we analyzed the protein levels and localization of mover in the adult mouse brain quantitatively, relative to the abundance of SVs. We found an unusually heterogeneous distribution, with high levels of mover in some regions, e.g., hippocampus and amygdala, and lower levels in other regions. Additionally, we analyzed the extent of colocalization of mover with vGluT1, a marker for excitatory synapses, and vGAT, a marker for inhibitory synapses. We discovered that also in respect to synapse types, mover shows a differential distribution. In particular, our study yielded three key observations:

First, mover is heterogeneously distributed among 16 brain regions. It is especially prominent in the VPa, SNu and the amygdala. Second, mover is heterogeneously distributed within the hippocampus. Mover levels, compared to average in the hemisphere, are particularly high in the stratum radiatum and oriens, while mover is absent from the pyramidal cell layers and the stratum lacunosum-moleculare. On a synapse level, in all subregions of the hippocampus tested, mover is present at excitatory synapses, and absent from inhibitory synapses. Third, unlike its distribution in the hippocampus, mover levels are homogeneously high throughout the amygdala. On a synapse level, mover is present at excitatory synapses in all subregions of the amygdala, but differentially distributed among inhibitory synapses: a high Pearson’s value for mover and vGAT (0.419) indicates the presence of mover at inhibitory synapses in the medioposterior nucleus, while low Pearson’s values indicate that mover is absent from most inhibitory synapses in the lateral and basolateral amygdala (0.162 and 0.140 respectively). Overall, our study reveals that mover is indeed differentially distributed among synapses in the adult mouse brain, and that its association with inhibitory synapses differs between brain regions and even within one brain region, i.e., the amygdala. These data raise the possibility that mover may act as a region-specific and synapse-specific regulator of synaptic transmission.

To determine the relative levels of mover protein we immunostained brain sections with a polyclonal mover antiserum used previously for western blotting, co-immunoprecipitation and immunogold labeling of SVs (Ahmed et al., 2013). Determining absolute protein amounts using this antibody is impossible with indirect immunofluorescence techniques. Confocal microscopy on the other hand allowed us to readily obtain a quantitative readout of the relative levels of mover in a large number of brain regions and its association with excitatory and inhibitory synapses. Therefore, this is the ideal technique for testing the hypothesis that mover is differentially distributed among brain regions and synapse types.

By using synaptophysin as a reference marker, we accounted for two principle caveats of our approach: first, the overall intensity of immunofluorescence varies between experiments. This is a general feature of immunofluorescence. Second, increased mover immunofluorescence in a certain brain region compared to its surroundings may represent increased levels of mover, or simply reflect an increased density of synapses or increased number of SVs per terminal in that region. We chose synaptophysin as a reference marker, as it is present at every synapse and likely on all SVs. Compared to using bassoon as a reference marker, synaptophysin allowed us to determine the abundance of mover relative to the number of SVs, as synaptophysin levels within a single synapse are directly proportional to the SVs content of this synapse (Navone et al., 1986). In contrast, staining for bassoon would only allow us to locate synapses, but would not yield any information on the amount of SVs in the synapse. Without a SV marker reference, increased mover levels at a certain synapse could be a trivial consequence of the synapse having more SVs. Thus, we used synaptophysin, a widely used general synapse marker, as our reference (Barak et al., 2010; Micheva et al., 2010). We cannot exclude the possibility that, unexpectedly, the abundance of synaptophysin itself is particularly high or low in certain brain areas. However, our immunostainings do not support this possibility, as the synaptophysin staining intensity was indeed rather uniform across the hemispheres, as expected. We conclude that by determining the ratio between mover and synaptophysin we can correct for differences in synapse density and synapse size in a certain brain area. In addition, comparing the ratio obtained for an individual brain region to the ratio in the whole hemisphere quantifies the abundance of mover relative to average in the hemisphere and corrects for differences in overall staining intensity between experiments. Note that we do not compare brain areas located at distinct levels along the rostro-caudal axis, because the average amount of mover differs at the different levels and depends on the number of regions with high mover intensity. For example, at a level with few high intensity regions, the lowest mover to synaptophysin ratio might be −25% compared to average, representing the complete absence of mover, while absence is represented by a value of −40% at a level with more high intensity regions. Comparing different levels is thus not meaningful. We therefore exclusively compare brain areas and their subregions at a certain level to the average of the hemisphere at the same level. Note that therefore our results apply to the indicated Bregma levels. Whether or not differences within one area of interest occur along the rostro-caudal axis was not determined. Using this stringent approach, we found that at the first level that we analyzed (the most rostral level; see Figure 1) mover was particularly abundant in islands of Calleja; at the second level, it was most abundant in the SNu and VPa; at the third level, it was highest in the amygdala; at the two most caudal levels, it was highest in the periaqueductal gray and the MLC. All levels except the level including the periaqueductal gray, SN and the VTA also contained regions where mover was below average, further emphasizing its heterogeneous distribution.

To quantify colocalization, we determined the Pearson’s correlation coefficient (further methodological discussion see Supplementary Information). Pearson’s coefficients for vGluT1 and vGAT were between 0.027 and 0.169, depending on the brain area and subregions. To apply a maximally stringent criterion, we used the Pearson’s value for vGluT1 and vGAT from each individual subregions as a threshold, and we considered mover as “not colocalized” with a marker when the Pearson’s value was the same or below the value for vGluT1 and vGAT in the same region. Using this criterion, mover is clearly present at excitatory synapses throughout the hippocampus and amygdala (Pearson’s values were between 0.359 and 0.713 for the subregions). In addition, mover is present at inhibitory synapses in the amygdala, with the highest colocalization with vGAT in the medioposterior amygdala (Pearson’s value of 0.419). In contrast, mover is clearly absent from inhibitory synapses in most subregions of the hippocampus, and likely absent in the stratum radiatum and oriens of the CA3, too. In these two CA3 layers, the Pearson’s coefficient for mover and vGAT was strikingly low (0.075 in the stratum radiatum and 0.044 in the stratum oriens). But since the Pearson’s coefficient for vGlut1 and vGAT was even lower in these regions (0.055 in the stratum radiatum and 0.027 in the stratum oriens) we cannot exclude that there is some degree of colocalization for mover with vGAT in these two areas. Note, however, that these Pearson’s values are very low, indicating that even in these regions only a small fraction of inhibitory synapses, if any, have mover. Overall, these data suggest that mover primarily regulates excitatory synaptic transmission in the hippocampus.

In the hippocampus, the abundance of mover varies strikingly among the layers, suggesting that mover may be particularly important for certain hippocampal synapses. Throughout the hippocampus, the levels of mover compared to synaptophysin are very low in the cell body layers, i.e., in the stratum granulosum of the dentate gyrus and the SPy of the CA3 and CA1. This is consistent with its absence from inhibitory terminals, which are arranged as perisomatic synapses in the cell body layers. Interestingly, mover levels are equally low in the outer molecular layer of the dentate gyrus and the stratum lacunosum-moleculare of the CA1. These are layers of the hippocampus that receive input from the entorhinal cortex, i.e., from outside the hippocampus. In contrast, mover levels compared to synaptophysin are most strikingly above average in the polymorph layer of the dentate gyrus, also called the hilus, as well as the stratum radiatum and the stratum oriens of the CA3 and CA1. These are three layers that contain axons and axon terminals arising from the principle cells of the hippocampus: the polymorph layer contains the glutamatergic axons of dentate gyrus granule cells, called mossy fibers. Collaterals of these mossy fibers make synapses within the polymorph layer, by targeting excitatory mossy cells and inhibitory basket cells. The mossy cell axons synapse in the inner molecular layer of the dentate gyrus, the basket cells synapse on the granule cell somata. Thus, through mossy fibers collaterals, dentate gyrus granule cells trigger both excitatory and inhibitory feedback onto themselves. The high levels of mover relative to synaptophysin in the polymorph region suggest a role for mover in mossy fiber axon collaterals, i.e., in presynaptic terminals targeting basket cells or mossy cells. In each case, mover would be important for the regulation of feedback loops within the dentate gyrus. The stratum radiatum and oriens contain the glutamatergic axons of the hippocampal pyramidal cells: these axons are called associational/commissural fibers when they target pyramidal cell dendrites within either CA3 or CA1, and they are called Schaffer collaterals when they run from the CA3 to the CA1 region. The fact that mover levels compared to synaptophysin are high in these regions, while they are low in the outer molecular layer of the dentate gyrus and the stratum lacunosum-moleculare, suggest that mover may be more important for the regulation of intra-hippocampal information flow and processing than for the entry of signals into the dentate gyrus and hippocampus.

Mover is present in mossy fiber terminals in the stratum lucidum of the CA3 (Kremer et al., 2007), and its abundance is slightly above average in these synapses, as indicated by our current study. Mossy fiber terminals are specialized nerve endings with a low release probability and strong capacity for facilitation (Nicoll and Schmitz, 2005). Their plasticity is regulated by an evolutionarily conserved presynaptic protein called tomosyn: knockdown of tomosyn in mossy fibers reduces presynaptic short-term and long-term potentiation, presumably by increasing basal release probability (Ben-Simon et al., 2015). Tomosyn is present in mossy fiber terminals, but absent from inhibitory terminals (Barak et al., 2010). The absence of mover from inhibitory terminals and presence at mossy fiber terminals is reminiscent of the distribution of tomosyn. In addition, mover regulated release probability and short-term plasticity at the calyx of Held, a specialized axo-somatic synapse located in the brainstem: knockdown of mover at the calyx of Held increases short-term depression and release probability (Körber et al., 2015). If mover had a similar role at mossy fiber terminals, it could add a vertebrate-specific function in regulating presynaptic plasticity to the established role of the conserved protein tomosyn. Knockout studies should throw light on the function of mover at these specialized synapses, and on its potential role for spatial learning and memory.

A similar layer-specific distribution of mover was detectable in neocortical fields, such as the S1. The S1, also called barrel cortex, is a highly organized structure that mediates touch and pain sensation from the whisker pad. The barrels are organized in columns and rows (Schubert et al., 2007). Whiskers are represented somatotopically, meaning that one barrel in a row represents the corresponding whisker in that row in the whisker pad (Welker, 1971; Welker and Woolsey, 1974). Input into the barrel cortex originates from two distinct thalamic nuclei, ventral posteromedial nucleus (VPm) and the posteromedial nucleus (POm). Fibers from the VPm form the lemniscal pathway and mainly project to the barrels in layer IV, while fibers from POm, which form the paralemniscal pathway, target layer Va pyramidal cells and layer I neurons to a smaller extent (Bosman et al., 2011). While the touch-mediating function of the lemniscal pathway has been described in detail (e.g., Nicolelis, 2005; Yu et al., 2006), the whole extent of functions of the paralemniscal pathway remains unclear, ranging from modulation of the lemniscal pathway (Ahissar et al., 2000) to pain sensation (Frangeul et al., 2014). Our stainings revealed higher mover intensities in layer V of the S1 and lower mover intensity in layer IV. This suggests a pathway-specific expression of mover in the paralemniscal pathway, which can potentially be used as a marker specific for this pathway, something that has been missing so far. In future experiments it remains to be seen whether mover also exhibits a pathway-specific function, such as modulating pain sensation or fine-tuning of touch sensation. Functional experiments, such as electrophysiological measurements and (*in vivo*) calcium imaging should shed light on this question and determine mover’s function in this pathway.

Unlike its heterogeneous distribution within the hippocampus and the neocortex, mover levels were homogeneously high, i.e., above average of the hemisphere, in the amygdala. Input into the different nuclei is diverse, just like the function of the amygdala. It has been connected to fear conditioning (LeDoux et al., 1990) hormone secretion (Eleftheriou and Zolovick, 1967) and emotional and sexual behavior (Kondo, 1992). In humans, the amygdala has even been described to be involved in psychiatric disorders, such as posttraumatic stress disorder (PTSD; Mahan and Ressler, 2012). The amygdala has been described as a very plastic structure (Sangha, 2015). The medial amygdaloid nucleus receives input from the main and accessory olfactory bulbs, which is involved in mediating socio-sexual behavior (Fernandez-Fewell and Meredith, 1994). Within this nucleus, glutamatergic and GABAergic neurons can be found. Both cell types project to the hypothalamus, but there is a subpopulation of GABAergic neurons which function as local circuit interneurons and most likely provide feedforward inhibition onto the excitatory neurons in the medioposterior amygdala. The high abundance of mover and high Pearson’s values for colocalization with vGAT suggest that mover might be involved in this local circuitry, shaping the input from the olfactory bulb to the medial amygdala and its output to the hypothalamus.

Most strikingly, mover is associated with inhibitory synapses in the amygdala, while it is absent from inhibitory synapses in the hippocampus. This raises the possibility that mover may act as a regulator of synapse function and plasticity with particular importance for the heterogeneity of inhibitory synapses and may thus contribute to a proper excitation-inhibition balance.

Except for its association with the hippocampus and amygdala, mover was especially abundant in the VPa and SNu of the adult mouse brain. Both areas have been connected to reward and reinforcement, and the VPa has also been suggested to be involved in addiction. High levels of mover in these regions could suggest a role for mover in these processes and warrant further investigation of mover’s role in reward and addiction.

We found mover as an interaction partner for the active zone scaffolding protein bassoon in a yeast-2-hybrid assay (Kremer et al., 2007) and later showed that it is associated with SVs (Ahmed et al., 2013). Interestingly, probing purified SVs with immunogold electron microscopy, mover was associated with only 16 percent of the SVs, while synaptophysin was associated with virtually all SVs (Ahmed et al., 2013). The strong association of synaptophysin with SVs lends further support to our assumption that synaptophysin is a faithful marker for SVs. The association of mover with only a fraction of purified SVs may indicate that mover is selectively attached to a subset of SVs in a given synapse, but it is also consistent with our observation that some synapses have very low levels of mover, or even no mover.

Mover and bassoon are two of a remarkably small number of proteins that are not evolutionarily conserved but rather evolved in a manner unique to vertebrates. Among these proteins are the active zone scaffolding piccolo, the motor adaptor syntabulin and the SV protein synuclein (George, 2002; Cai et al., 2007; Gundelfinger et al., 2016). Whether these proteins confer certain vertebrate-specific functions to the conserved core machinery of neurotransmitter release is an open question. Double knockdown of bassoon and piccolo leads to disassembly of synapses, suggesting that these two multi-domain scaffolding proteins stabilize vertebrate synapses (Waites et al., 2013). Other vertebrate-specific proteins are thought to increase the functional heterogeneity of synapses in the brain (Emes et al., 2008; Ryan and Grant, 2009). A heterogeneous expression, such as that revealed for mover here, is expected as a key feature of such modulatory proteins. At the calyx of Held synapse, short term depression and release probability are increased after knockdown of mover, suggesting that at least one of the roles of mover is to regulate release probability and short-term plasticity (Körber et al., 2015). Release probability was also increased at the endbulb of Held synapse in mice expressing mutant bassoon (Mendoza Schulz et al., 2014). In contrast, release probability was unaffected in bassoon knockout mice at the cerebellar mossy fiber synapse, while SV reloading was impaired (Hallermann et al., 2010). Our observation that mover is heterogeneously expressed, in combination with the interaction of mover with bassoon (Kremer et al., 2007) and its association with a subset of SVs (Ahmed et al., 2013), raises the possibility that the levels of mover may regulate the interaction of bassoon with SVs at active zones, thus contributing to presynaptic heterogeneity. Functional studies involving knock-out and knock-in models of mover, employing electrophysiological and biochemical methods, are required to analyze the presynaptic pathways modulated by mover.

While high mover abundance could indicate crucial mover functions, regions with low expression levels of mover should not be disregarded. Mover abundance in the ACC, for example, was below average in the adult mouse brain. However, in human schizophrenic patients, mover has been shown to be upregulated in the ACC (Clark et al., 2006), raising the possibility that mover may be regulated by neuronal activity. For example, aberrant neuronal activity associated with schizophrenia could upregulate mover. If mover dampens presynaptic release, as suggested by knockdown at the calyx of Held (Körber et al., 2015), its activity-dependent upregulation could occur as a protective mechanism to confine runaway excitation.

Further studies should reveal whether activity-dependent expression contributes to the remarkably heterogeneous distribution of mover. In any case, its differential association with synapses on the level of brain areas, subregions and types of synapses renders it a candidate for a protein that generates synaptic heterogeneity.

## Author Contributions

RW performed experiments, analyzed data, contributed to experimental design and prepared the manuscript. TD designed the studies and the entire project and also prepared the manuscript. Both authors discussed the results, read and approved the final manuscript.

## Conflict of Interest Statement

The authors declare that the research was conducted in the absence of any commercial or financial relationships that could be construed as a potential conflict of interest.
