# Supplementary material for: The Presynaptic Protein Mover Is Differentially Expressed Across Brain Areas and Synapse Types
Source: Front Neuroanat. 2018 Jul 13;12:58. doi: 10.3389/fnana.2018.00058 (PMC6053503; doi:10.3389/fnana.2018.00058)
Supplement: Supplementary file 1 [file Image_1.PDF]

The Presynaptic Protein Mover is Differentially Expressed across Brain Areas and Synapse Types

Rebecca Wallrafen and Thomas Dresbach

Supplementary Information

Supplementary method discussion

Pearson's coefficient measures the linear correlation between two variables X and Y, which in our case are the immunofluorescence signal of protein 1, i.e. mover, and that of protein 2, i.e. vGluT1 or vGAT. The Pearson's coefficient can range between +1 (total positive linear correlation) and -1 (total negative correlation). A value of 0 represents no correlation between the variables. Theoretically, values of the Pearson's coefficient should be close to -1 when analyzing vGluT1 and vGAT, as their immunosignals avoid each other. In a scenario where there were exclusively either glutamatergic or GABAergic synapses, and nothing else (i.e. no space occupied by, for example, cholinergic, dopaminergic synapses or astrocytes), the values would range around -1. However, given that some space is occupied by sites lacking both vGluT1 and vGAT, Pearson's values around 0-0.1 are expected, as double-absence is also positive correlation.

Supplementary results

To quantify colocalization we determined the Pearson's correlation coefficient. This coefficient measures the linear correlation between two variables X and Y, which in our case are the immunofluorescence signals of two different proteins, i.e. vGluT1 and vGAT. The Pearson's coefficient can range between +1 (total positive linear correlation) and -1 (total negative correlation). A value of 0 represent no linear correlation between the variables. Theoretically, values of Pearson's coefficient should be close to -1 when analysing vGluT1 and vGAT, as their immunosignals avoid each other. In a scenario where there were exclusively either glutamatergic or GABAergic synapses, and nothing else (i.e. no space occupied by, for example, cholinergic, dopaminergic synapses or astrocytes), the values would range around -1. However, given than some space is occupied by sites lacking both vGluT1 and vGAT, Pearson's values around 0-0.1 are expected, as double-absence is also positive correlation. This is indeed what we found (see **Figure S1** and **S3**): Pearson's coefficients for vGluT1 and vGAT were between 0.027 and 0.169. The rather high values in the polymorph layer of DG (0.169) and stratum lucidum of CA3 (0.103) can be explained by the complex intermingling of GABAergic synapses and the extraordinarily large mossy fiber terminals in these regions, but still reflect lack of colocalization. We therefore conclude that Pearson's values between mover and any marker of less than 0.17 can be regarded as no colocalization.

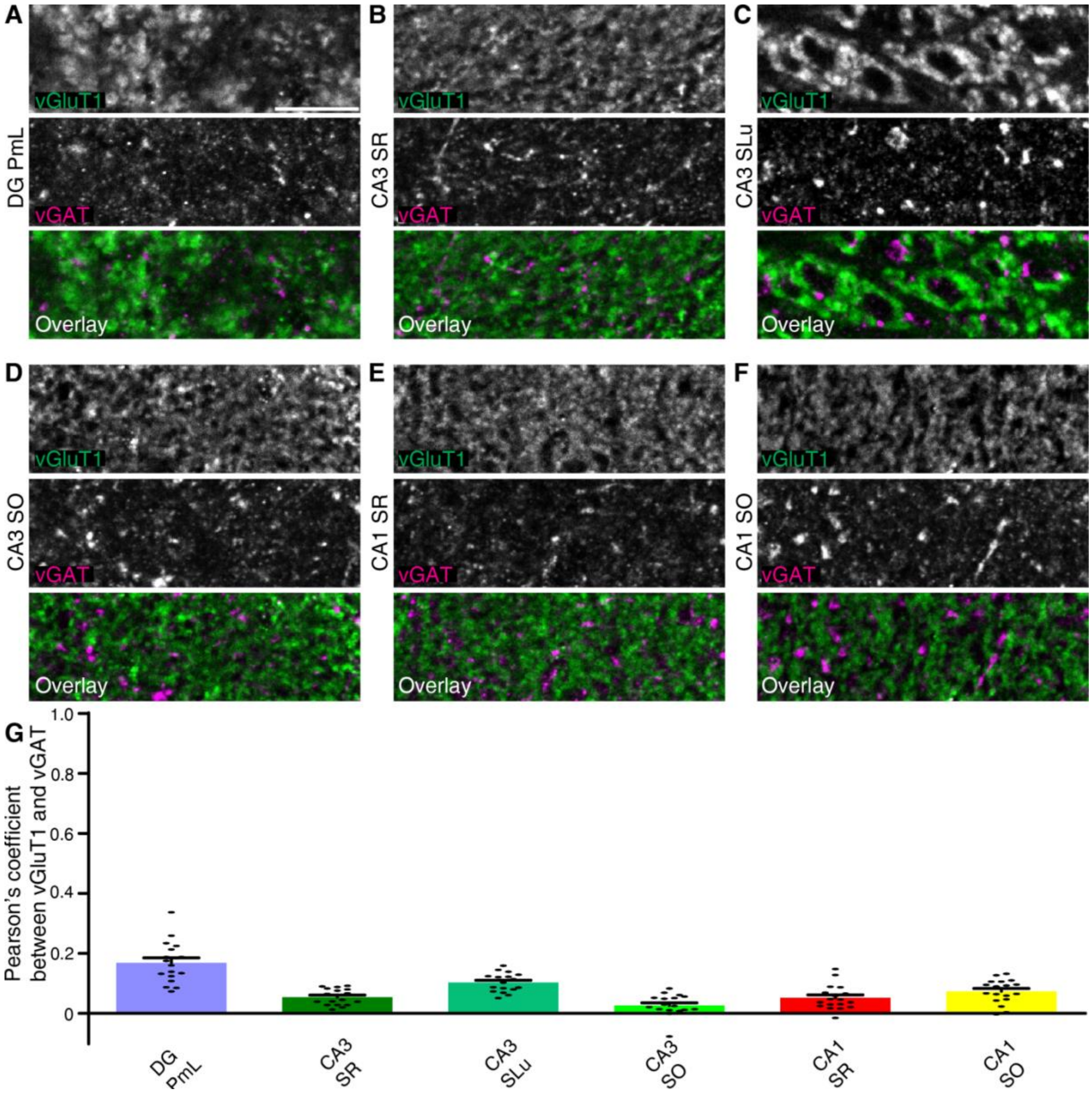

Figure S1: Colocalization between vGluT1 and vGAT in the hippocampus. Pearson's correlation coefficient was determined for colocalization between vGluT1 (green, upper panel) and vGAT (magenta, lower panel) in areas of the hippocampus with high mover to synaptophysin ratio. The overlay of the two stainings is shown in the lower panel. Representative distribution of the two proteins in the polymorph layer of the DG (Pearson's correlation coefficient 0.169, (**A**)), stratum radiatum of CA3 (Pearson's correlation coefficient 0.055, (**B**)), stratum lucidum of CA3 (Pearson's correlation coefficient 0.103, (**C**)), stratum oriens of CA3 (Pearson's correlation coefficient 0.027, (**D**)), stratum radiatum of CA1 (Pearson's correlation coefficient 0.052, (E)), stratum oriens of CA1 (Pearson's correlation coefficient 0.074, (**F**)). Visualization of the Pearson's correlation coefficients between vGluT1 and vGAT in the different regions (**G**). Bars show average ± SEM. PmL, polymorph layer of dentate gyrus; SR, stratum radiatum (of either CA3 or CA1); SLu, stratum lucidum of CA3; SO, stratum oriens (of either CA3 or CA1). Scale bar = 10µm.

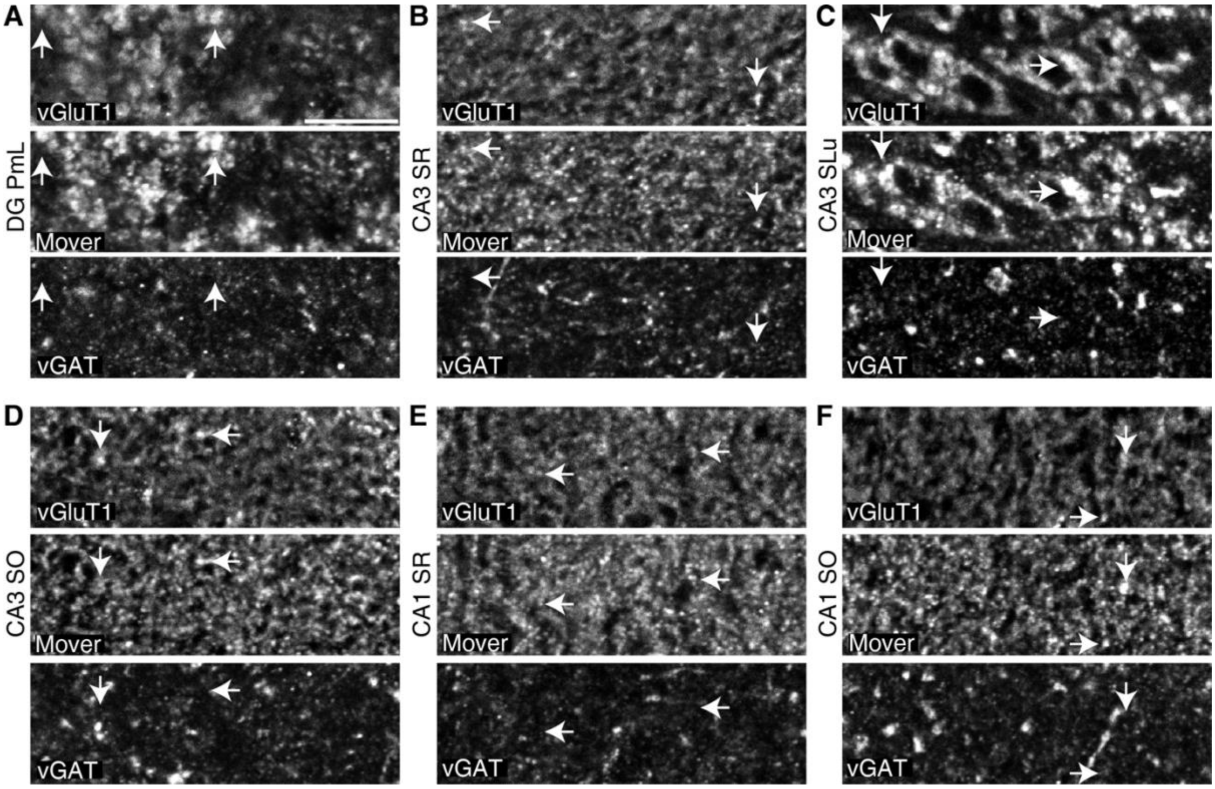

Figure S2: Mover colocalization with presynaptic markers vGlut1 and vGAT in the mouse hippocampus. Black and white images of the panels shown in Fig. 3 for additional visualization of colocalization between Mover (middle panel) and vGluT1 (upper panel) or vGAT (lower panel). Arrows indicate areas of colocalization between mover and vGluT1 (but not vGAT), arrowheads show areas of colocalization between mover and vGAT (but not vGluT1) in the polymorph layer of the DG (**A**), stratum radiatum of CA3 (**B**), stratum lucidum of CA3 (**C**), stratum oriens of CA3 (**D**), stratum radiatum of CA1 (**E**) and stratum oriens of CA1 (**F**). PmL, polymorph layer of dentate gyrus; SR, stratum radiatum (of either CA3 or CA1); SLu, stratum lucidum of CA3; SO, stratum oriens (of either CA3 or CA1). Scale bar = 10μm.

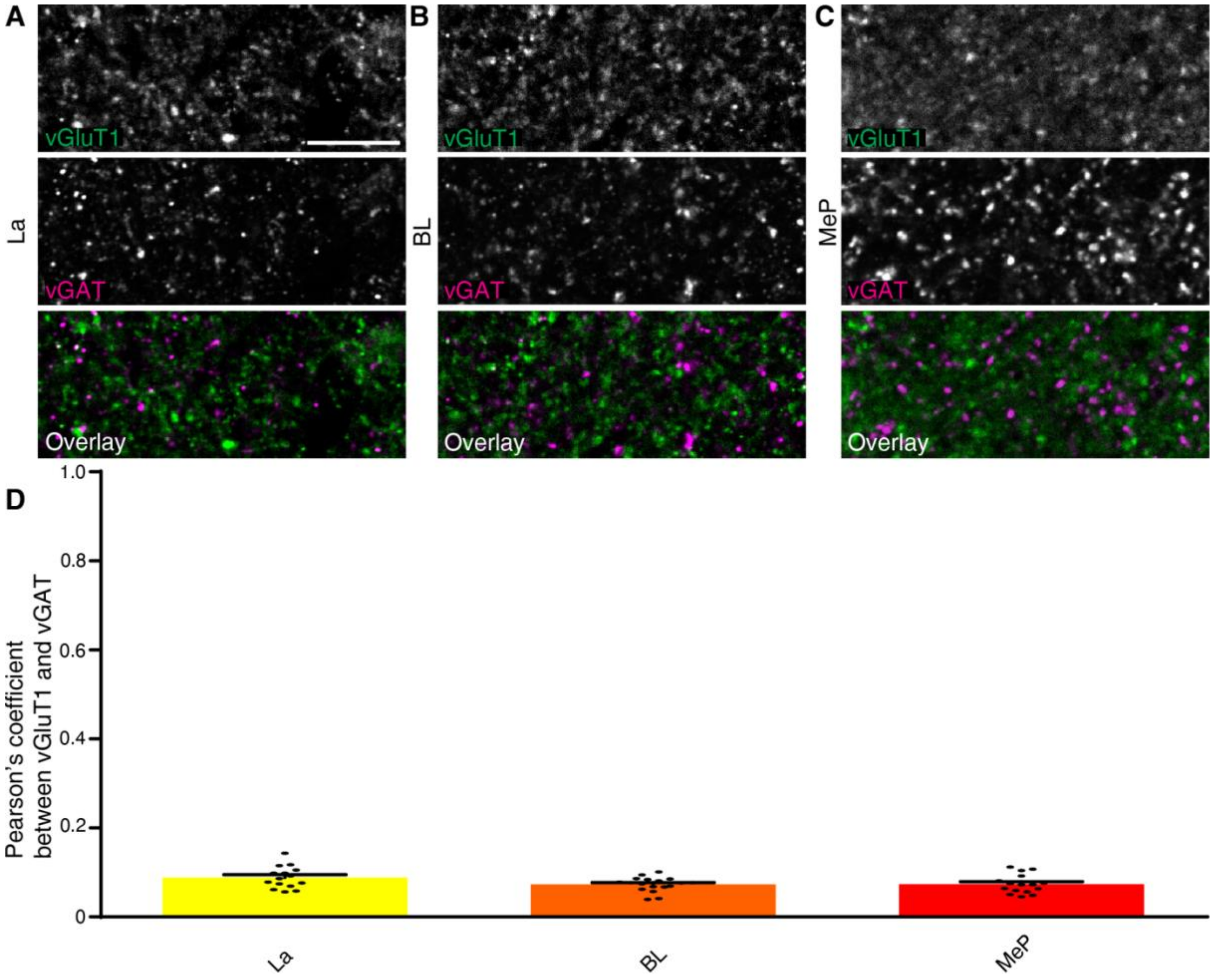

Figure S3: Colocalization between vGluT1 and vGAT in the amygdala. Pearson's correlation coefficient was determined for colocalization between vGluT1 (green, upper panel) and vGAT (magenta, lower panel) in the different nuclei of the amygdala. The overlay of the two stainings is shown in the lower panel. Representative distribution of the two proteins in the lateral (Pearson's correlation coefficient 0.088, (**A**)), basolateral (Pearson's correlation coefficient 0.073, (**B**)), and medioposterior nuclei (Pearson's correlation coefficient 0.074, (**C**)). Visualization of the Pearson's correlation coefficients between vGluT1 and vGAT in the different regions (**D**). Bars show average ± SEM. La, lateral nuclei of the amygdala; BL, basolateral nuclei of the amygdala; MeP, medioposterior nuclei of the amygdala. Scale bar = 10µm.

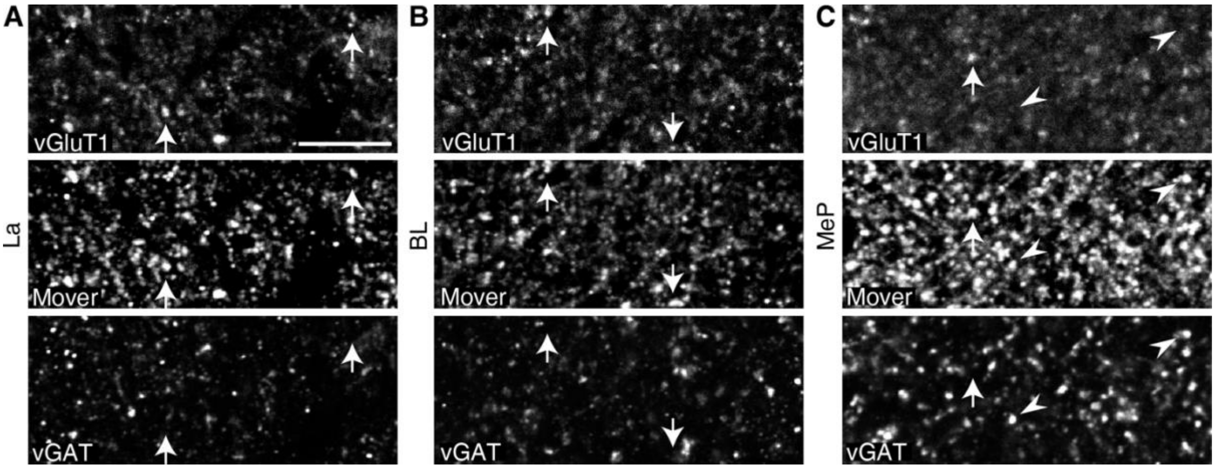

Figure S4: Mover colocalization with presynaptic markers vGlut1 and vGAT in the mouse amygdala. Black and white images of the panels shown in Fig. 5 for additional visualization of colocalization between Mover (middle panel) and vGluT1 (upper panel) or vGAT (lower panel). Arrows indicate areas of colocalization between mover and vGluT1 (but not vGAT), arrowheads show areas of colocalization between mover and vGAT (but not vGluT1) in the lateral (**A**), basolateral (**B**) and medioposterior nuclei (**C**) of the amygdala. La, lateral nuclei of the amygdala; BL, basolateral nuclei of the amygdala; MeP, medioposterior nuclei of the amygdala. Scale bar = 10μm.
